# Supplementary material for: The Systems Biology Research Tool: evolvable open-source software
Source: BMC Syst Biol. 2008 Jun 29;2:55. doi: 10.1186/1752-0509-2-55 (PMC2446383; doi:10.1186/1752-0509-2-55)
Supplement: Additional file 1 — SBRT Archive. An archive of the current version of the Systems Biology Research Tool. [file 1752-0509-2-55-S1.zip › sbrt-1.4.0/doc/developers_guide/api/sbrt/kernel/fba/proc/RandomObjectiveGenerator.html]

RandomObjectiveGenerator


|  |  |  |
| --- | --- | --- |
| **Overview** \| **Package** \| **Class** \| **Use** \| **Tree** \| **Deprecated** \| **Index** \| **Help** | |  |
| **PREV CLASS**   **NEXT CLASS** | **FRAMES**    **NO FRAMES**     **All Classes** |  |
| SUMMARY: NESTED \| FIELD \| CONSTR \| METHOD | DETAIL: FIELD \| CONSTR \| METHOD |  |


---


## sbrt.kernel.fba.proc Class RandomObjectiveGenerator

```
java.lang.Object
  sbrt.kernel.fba.proc.FbaProcess<LinearComb<java.lang.String>>
      sbrt.kernel.fba.proc.RandomObjectiveGenerator
```

**All Implemented Interfaces:**: KernelProcess<LinearComb<java.lang.String>>, MonitoredProcess<LinearComb<java.lang.String>>

---

``` public final class RandomObjectiveGenerator extends FbaProcess<LinearComb<java.lang.String>> implements MonitoredProcess<LinearComb<java.lang.String>> ```

This class is used to generate random objective functions.

**Author:**
:   This class was written and documented by Jeremiah Wright
    while in the Wagner lab.

---

**Constructor Summary**

`RandomObjectiveGenerator(Fluxome fluxome)`
  
          Constructs a new random objective function generator.


| **Method Summary** | |
| --- | --- |
| `Fluxome` | `getFluxome()`             Returns the fluxome for which random objective functions are to be generated. |
| `ProgressMonitor` | `getProgress()`             Returns a progress monitor for this process. |
| `void` | `run(ResultsManager<LinearComb<java.lang.String>> resultsManager)`             Generates random objective functions and adds them to the provided results manager. |
| `void` | `setIterations(int iterations)`             Sets the number of random objective functions to be produced. |
| `void` | `setRandom(java.util.Random random)`             Sets the random number generator to the one provided. |

**Methods inherited from class sbrt.kernel.fba.proc.FbaProcess**

`toString`

**Methods inherited from class java.lang.Object**

`clone, equals, finalize, getClass, hashCode, notify, notifyAll, wait, wait, wait`

**Constructor Detail**

### RandomObjectiveGenerator

```
public RandomObjectiveGenerator(Fluxome fluxome)
```

:   Constructs a new random objective function generator.

    **Parameters:**: `fluxome` - the fluxome for which random objective functions are to be generated.


**Method Detail**

### getFluxome

```
public Fluxome getFluxome()
```

:   Returns the fluxome for which random objective functions are to be
    generated.

    :   **Specified by:**: `getFluxome` in class `FbaProcess<LinearComb<java.lang.String>>`
    :   **Returns:**: the fluxome for which random objective functions are to be generated.

---


### getProgress

```
public ProgressMonitor getProgress()
```

:   Returns a progress monitor for this process.

    :   **Specified by:**: `getProgress` in interface `MonitoredProcess<LinearComb<java.lang.String>>`
    :   **Returns:**: a progress monitor for this process.

---


### setRandom

```
public void setRandom(java.util.Random random)
```

:   Sets the random number generator to the one provided.

    :   **Parameters:**: `random` - the random number generator to use.

---


### setIterations

```
public void setIterations(int iterations)
```

:   Sets the number of random objective functions to be produced.

    :   **Parameters:**: `iterations` - the number of random objective functions to be produced.

---


### run

```
public void run(ResultsManager<LinearComb<java.lang.String>> resultsManager)
```

:   Generates random objective functions and adds them to the provided
    results manager.

    :   **Specified by:**: `run` in interface `KernelProcess<LinearComb<java.lang.String>>`
    :   **Parameters:**: `resultsManager` - the results manager to which the random objective functions will be added.


---


|  |  |  |
| --- | --- | --- |
| **Overview** \| **Package** \| **Class** \| **Use** \| **Tree** \| **Deprecated** \| **Index** \| **Help** | |  |
| **PREV CLASS**   **NEXT CLASS** | **FRAMES**    **NO FRAMES**     **All Classes** |  |
| SUMMARY: NESTED \| FIELD \| CONSTR \| METHOD | DETAIL: FIELD \| CONSTR \| METHOD |  |


---
